# A Challenging Diagnosis: Placental Mesenchymal Dysplasia—Literature Review and Case Report

**DOI:** 10.3390/diagnostics12020293

**Published:** 2022-01-24

**Authors:** Claudia Mehedintu, Francesca Frincu, Oana-Maria Ionescu, Monica Mihaela Cirstoiu, Maria Sajin, Maria Olinca, Elvira Bratila, Aida Petca, Andreea Carp-Veliscu

**Affiliations:** 1 Department of Obstetrics and Gynecology, “Carol Davila” University of Medicine and Pharmacy, 020021 Bucharest, Romania; claudia.mehedintu@umfcd.ro (C.M.); ionescuoanamaria@gmail.com (O.-M.I.); monica.cirstoiu@umfcd.ro (M.M.C.); elvira.bratila@umfcd.ro (E.B.); aida.petca@umfcd.ro (A.P.); andreea_veliscu@yahoo.com (A.C.-V.); 2 Department of Pathology, “Carol Davila” University of Medicine and Pharmacy, 020021 Bucharest, Romania; maria.sajin@umfcd.ro (M.S.); maria.olinca@umfcd.ro (M.O.)

**Keywords:** placental mesenchymal dysplasia, molar pregnancy, alpha-fetoprotein, β-human chorionic gonadotropin

## Abstract

We describe a 22-year-old woman (2-gravid) case who was referred to our clinic at 18 weeks of gestation for a placenta with vesicular lesions discovered on prenatal examination routine. An ultrasound exam at 31 weeks of gestation showed numerous vesicular lesions, which gradually augmented as the pregnancy advanced. A live normal-appearing fetus was confirmed by intrauterine growth restriction (IUGR). The maternal serum β-human chorionic gonadotropin level remained in normal ranges. At some point, a multidisciplinary medical consensus considered the termination of the pregnancy, but the patient refused to comply. At 33 weeks of gestation, preterm premature rupture of membranes (pPROM) occurred, and she spontaneously delivered a 1600 g healthy female baby with a good long-term outcome. Placental mesenchymal dysplasia (PMD) was retrospectively diagnosed after confronting the data from ultrasound, chorionic villus sampling (CVS), amniocentesis, pathological examination, and immunohistochemical stain. The lack of sufficient reports of PMD determines doctors to be cautious and reserved, approaching these cases more radically than necessary. We reviewed this disease and searched for all cases of PMD associated with healthy, live newborns.

## 1. Introduction

The first case of placental mesenchymal dysplasia (PMD) was in 1991, as mesenchymal hyperplasia of the stem villi in the placenta leading to increased placental volume and exhibiting high levels of maternal serum alpha-fetoprotein [1,2]. Although the reported PMD incidence is around 0.02% of all pregnancies, with a female-to-male sex ratio of 3.6–4.0: 1 [3,4,5], the exact incidence is, unfortunately, unknown, as this rare clinical entity is often mistaken for molar pregnancy due to their similar ultrasound features [6]. Most of the PMD cases correlate with intrauterine fetal death (IUFD), intrauterine growth restriction (IUGR), Beckwith–Wiedemann syndrome (BWS), or preeclampsia, but in rare cases, it can associate a normal fetus pregnancy [3,7,8]. Fetal karyotyping is mandatory and is usually euploid, although patients with aneuploidy have been reported [1]. Pathologically, PMD is characterized by mesenchymal hyperplasia, aneurysmal dilatation of chorionic vessels, edema of the stem-cell villi, and the absence of trophoblastic proliferation [1,2,8].

Due to the lack of sufficient reports of PMD, the physician’s attitude towards such cases is reserved and cautious. The most challenging differential diagnosis to ascertain is the hydatidiform mole, which requires an extreme therapeutic attitude, with pregnancy termination and post-abortion treatment with methotrexate. Consequently, the lack of knowledge about this pathology leads doctors to approach cases radically, similar to a hydatidiform mole. As far as we know, this is the first review to bring together all cases published about healthy live newborns coming from PMD pregnancies. This review is necessary to shed light on this pathology for better management of such patients. It is also a resourceful material to bring more knowledge, so every specialist considers such a diagnosis in their practice. Undoubtedly, if we know what we are looking for, we may find it. We present the case of an initially misdiagnosed PMD with a normal fetus.

## 2. Materials and Method

PubMed was searched from its inception until 16 December 2021 for all studies on placental mesenchymal dysplasia. We focused our search on pathogenic mechanisms, genetics, associated maladies, fetal and maternal outcomes. We used “MeSH” (PubMed) terms and free text words.

Our search included all study types for the review section to be most comprehensive. Thus, 176 articles were screened. Only case reports and case series of PMD with healthy neonates were included in the results section. We evaluated 104 articles and excluded literature that contained within the title or the abstract terms like abortion, in utero fetal death, stillbirth, fetuses or neonates with morphological abnormalities, genetic syndromes (including Beckwith–Wiedeman Syndrome), intellectual disability, and a poor outcome at follow-up. We included only the histopathologically confirmed PMD with normal, healthy babies. In the end, 41 articles were comprised, totaling 69 newborns without morphological abnormalities or genetic disorders, born from pregnancies with PMD. We analyzed only English literature.

## 3. Case Report

**Figure 1:**
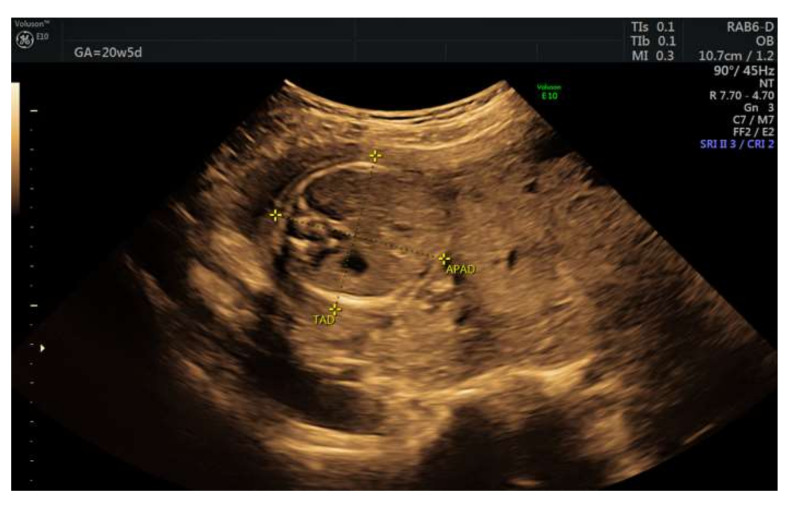
Placentomegaly with severe oligohydramnios.

**Figure 2:**
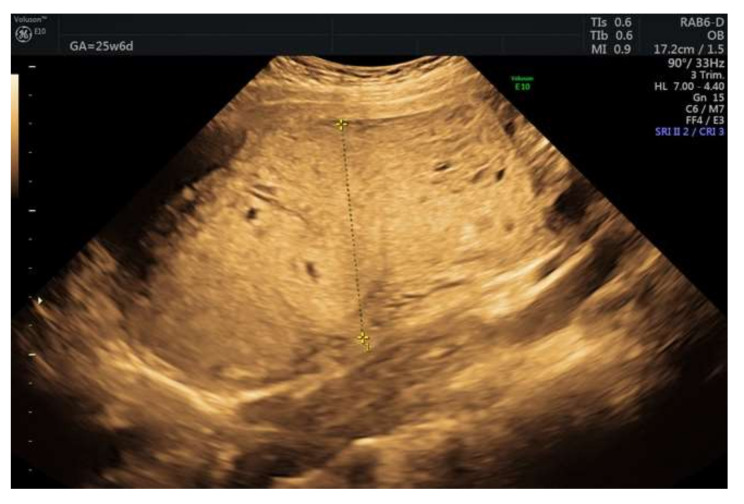
“Swiss-cheese” or “Moth-eaten” appearance of the placenta.

A 22-year-old pregnant woman was referred to our service for further investigation due to a low amount of amniotic fluid (single amniotic fluid pocket of 2.77 cm) at the 18-week routine scan. The patient underwent a first-trimester screening scan at 13 weeks of gestation and showed a unique live fetus with normal fetal morphology, a nuchal thickness of 2.2 mm, a beta hCG in normal ranges, and a low risk for IUGR. Toxoplasmosis, rubella cytomegalovirus, herpes simplex, and HIV (TORCH) complex, Listeria antibodies, and complementary tests were within normal limits. She previously gave birth spontaneously at 39 weeks of gestation to a healthy normal-weight baby, Apgar score 10, with no signs of newborn asphyxia. Our evaluation excluded amniotic membrane rupture or renal abnormalities as the primary cause for the severe oligoamnios and showed a “swiss” characteristic of the placenta and normal weighted fetus (Figure 1 and Figure 2).

**Figure 3:**
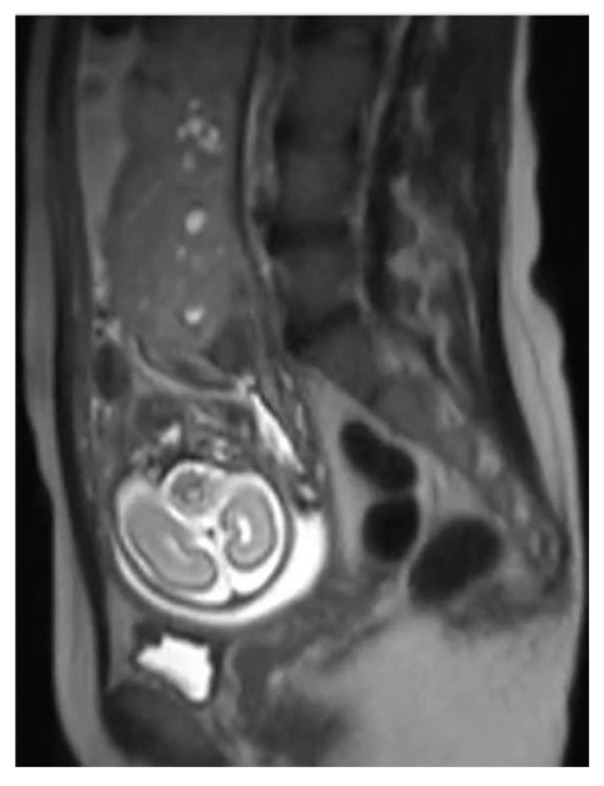
MRI scan showing placentomegaly, with no signs for abnormal adherence and the fetus head.

**Figure 4:**
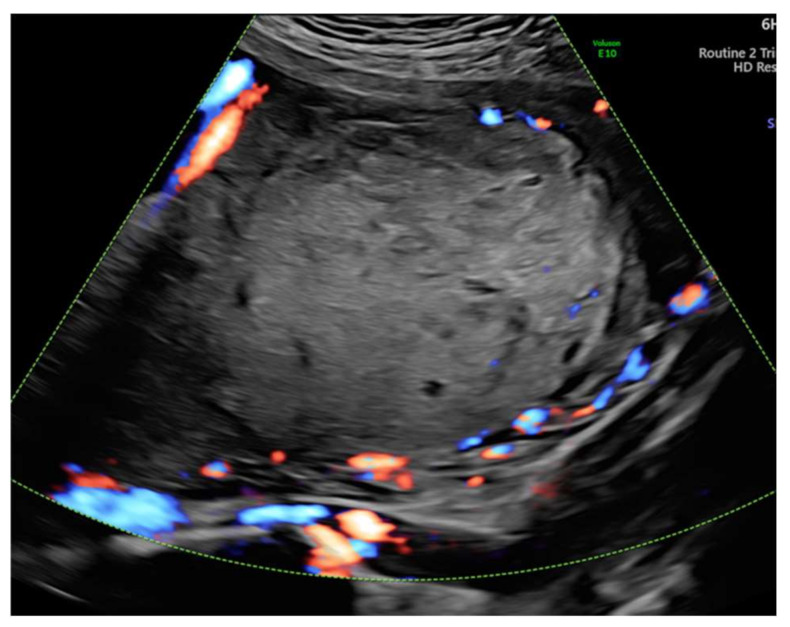
Doppler showed normal vascularization at 30 weeks of gestation.

The initial diagnosis was molar pregnancy with a normal fetus. We performed chorionic villus sampling (CVS), and the genetic analysis detected two genetic profiles at the level of the placenta, one with maternal and paternal normal alleles and one with paternal alleles only. Blood samples from both parents were collected (for comparative results). At 20 weeks of gestation, we performed amniocentesis that showed a normal female fetus in terms of chromosomes 21, 18, 13, and X. Given the complications of a molar pregnancy, the interdisciplinary team, which included a geneticist, a maternal-fetal medicine specialist, and an oncologist considered the termination of pregnancy. The patient refused pregnancy termination and insisted on further monitoring. All this time, the serum beta hCG level remained in the normal range (49,649 min/mL at 20 weeks and 77,070 mUI/mL at 24 weeks). The 24-weeks ultrasound revealed an enlarged multicystic placenta and increased subplacental vascularity, suggestive of placenta accreta spectrum. We referred the patient to a magnetic resonance imaging (MRI) scan that revealed multiple cysts within the placenta and no signs of abnormal adherence (Figure 3). At 31 weeks, the fetal growth was equivalent to 29 weeks and two days. Doppler assessment showed normal fetal vascularity (Figure 4) and a short cervix (11 mm) with funneling.

**Figure 5:**
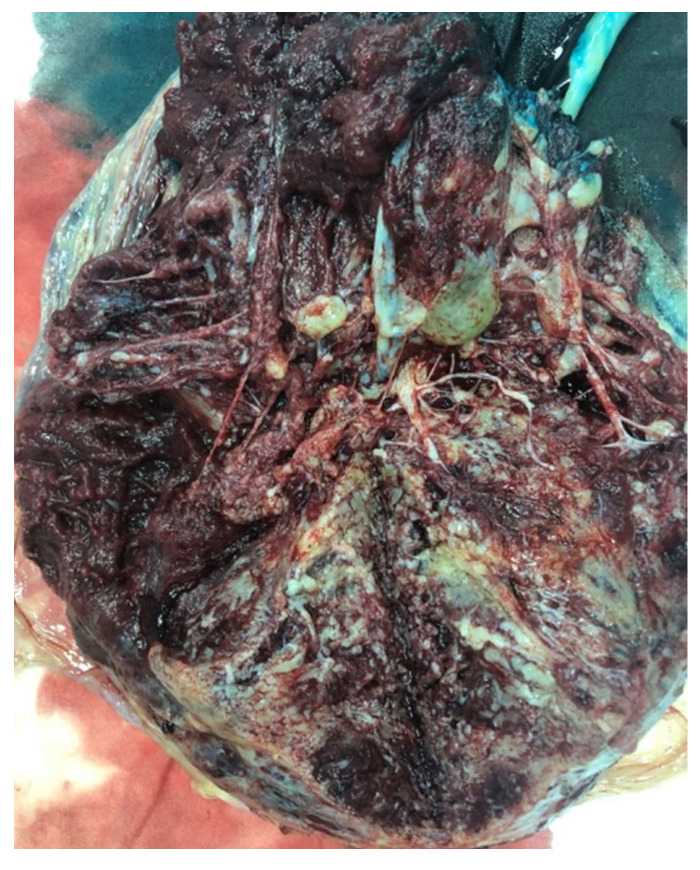
Placentomegaly with dilated vessels on the chorionic plate.

**Figure 6:**
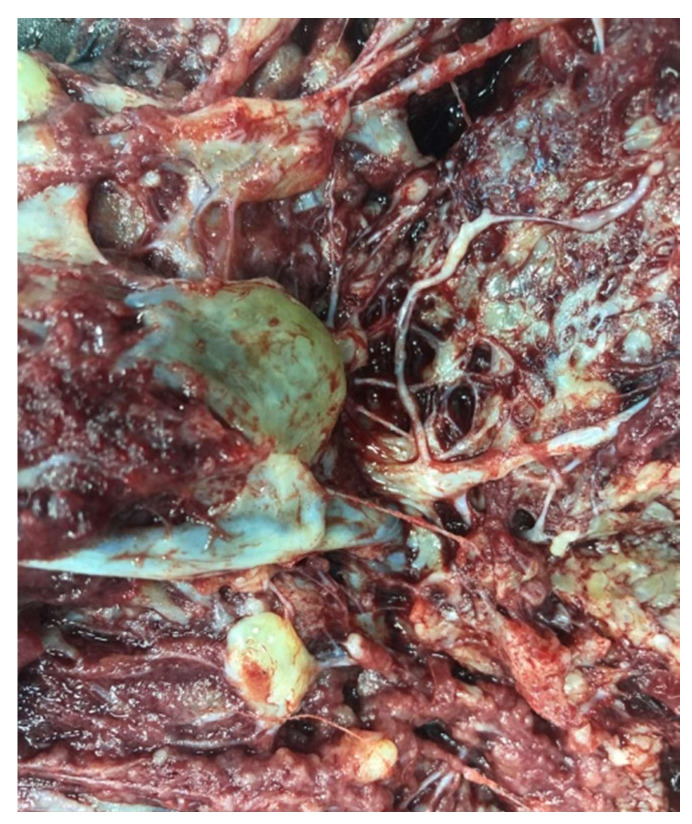
Hydropic cysts with features for partial hydatiform mole.

**Figure 7:**
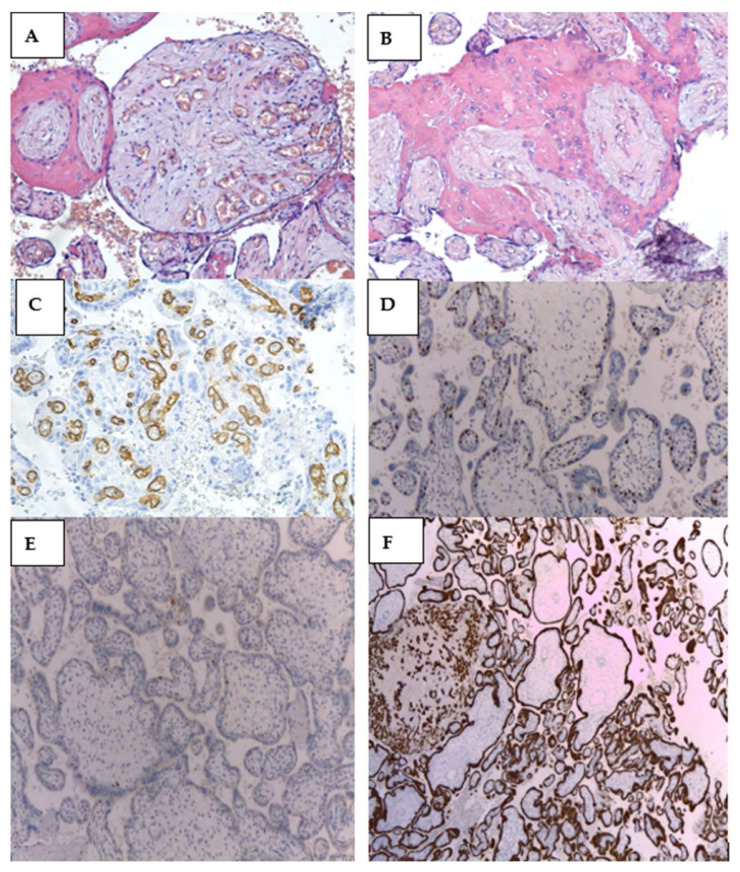
(**A**) Normal placental villi with intravillous vascularity present and villi with absent vascularity with perivillous fibrinoid. Perivillous hematic overflow HE, 200×;(**B**) Placental villi with absent intravillous vasculature and circumferential perivascular amyloid deposits. Affected syncytiotrophoblast with scattered cells among fibrinoid deposits, HE, 200×; (**C**) Normal placental villi, with normal capillary density. Immunohistochemical staining with anti-CD34 antibody (marks capillary endothelium in brown), 200×; (**D**) Normal p57 expression in the trophoblast, but absent stromal staining in placental mesenchymal dysplasia, IHC staining with DAB chromogen 10×; (**E**) p53 protein expressed in the nuclei of some trophoblastic cells (p53 wild-type pattern), IHC staining with DAB chromogen 10×; (**F**). Intense positive cytokeratin 7 staining of the villous trophoblastic cells, IHC staining with DAB chromogen 10×.

The patient gave birth spontaneously at 33 weeks of gestation to a 1600 g healthy female baby, Apgar 8/9. The placenta measured 24 × 18 × 6 cm and weighed 880 g. Gross examination suggested placentomegaly with features for partial hydatiform mole, vascular dilatation on the chorionic plate, and hydropic cysts (Figure 5 and Figure 6). Histopathological examination revealed edematous stem villi, with absent intravillous capillarity, surrounded by thick-walled dysplastic vessels and perivillous amyloid deposition, without atypical trophoblastic proliferation. On immunohistochemical stains, the cells that line the cistern appeared negative for CD-34. All findings were compatible with placental mesenchymal dysplasia (PMD). We performed an immunohistochemical panel using the standard procedure. Enzyme-conjugated for secondary antibodies were applied, and the specific staining was visualized after adding the enzyme-specific substrate. Immunohistochemistry for p57 shows staining of the stromal cells and villous cytotrophoblast. CD 34 and podoplanin showed multiple thick-walled blood vessels in the villi. We also investigated the placental tissue with an anti-p53 antibody, and the wild-type expression of this protein was documented (Figure 7).

The newborn had a favorable evolution, with no visceral or biochemical abnormalities (thrombopenia/ anemia).

## 4. Results

We present the data on PMD pregnancies cases with healthy fetuses. Both Table 1 and Table 2 illustrate the same cases reviewed in the literature. Each table describes the characteristics of the 69 healthy newborns from pregnancies with placental mesenchymal dysplasia. Table 1 illustrates the karyotype, beta hCG, and AFP levels, as well as the placental characteristics. Table 2 shows the fetal outcomes and the mother’s complications throughout the pregnancy.

Table 1 shows that all healthy fetuses from pregnancies with placental mesenchymal dysplasia had a normal karyotype, 46XX or 46XY, without specific abnormalities. In all cases, alpha-fetoprotein was elevated, unlike normal or hydatidiform mole pregnancies. In all cases, beta hCG was normal. Placenta mass was significantly increased compared to the median for the age of the pregnancy. Placental histopathological analysis revealed in all cases a mixture of normal-looking areas and numerous clusters of grape-like fluid-filled vesicles, swollen, myxoid stem villi and vesicles scattered throughout the parenchyma, multiple dilated chorionic plate vessels with areas of aneurysmal dilation, hypercapillarization, chorioanginosis, and hypertrophy of the vessels of the stem and subchorial villi, with aspects of thrombosis of the lumen. No placenta showed trophoblastic hyperplasia. Where data were available, all abnormal stromal villous cells were negative for CD 34, D 2–40, had absent or slightly detectable ki-67, and absent p57kip2. Of all cases, only one mother developed choriocarcinoma. The majority of the fetuses had IUGR. Fetal reported problems included fetal anemia, thrombocytopenia, physiological neonatal jaundice, and benign hepatic cysts. All babies presented good outcomes during the follow-up visits. Only a few cases were complicated with preeclampsia and gestational diabetes. We can not establish a connection between the placental issue and these conditions.

## 5. Discussions

We illustrated an initially misdiagnosed case of PMD with a healthy, live fetus.

PMD is a rare disorder with comparable ultrasound features with partial mole pregnancy or complete mole with a normal fetus coexisting. The necessity for an accurate prenatal diagnosis is undoubted since these conditions have a significantly different prognosis for both the fetus and mother [44].

When facing an enlarged multicystic placenta on ultrasound with a normal fetus, doctors must consider several elements: the maternal serum β-hCG, the maternal serum alpha-fetoprotein (MSAFP), ultrasound Doppler assessment of the blood flow within the placenta, and karyotype [12]. PMD exhibits normal or slightly increased beta hCG and high MSAFP [3]; however, 38% of cases of PMDs have an increase in beta hCG levels [45]. The high MSAFP—the principal characteristic of PMD—is the consequence of an increased surface for transfer due to the enlarged placenta and the increased permeability of thin-walled vessels within the stem villi [46,47]. Moreover, the degree of adverse fetal outcomes might correlate with the MSAFP levels [46]; even in the absence of PMD, approximately 10% of fetuses carried by women with a persisting elevated AFP level ≥2.5 Multiple of the Median (MoM), die in utero [9]. In PMD, the fetal erythropoiesis might not adjust to the sharp increase in the vascular plate, leading to fetal anemia or adverse outcomes, such as fetal growth restriction, hydrops fetalis, or intrauterine fetal death (IUFD) [9,46]. In these cases, intensive monitoring of the Middle Cerebral Artery-Peak Systolic Velocity (MCA-PSV) using ultrasound may be helpful to avoid IUFD in some PMD pregnancies [9].

Ultrasound is currently the most suitable instrument for examining the placenta from the beginning of the pregnancy and detecting PMD [7]. The classic sonographic characteristic is a thickened placenta with multiple hypoechoic spaces due to dilation of chorionic vessels: “swiss-cheese” or “moth-eaten placenta” [48,49]. Although Umazume et al., reported a case of PMD with placental cystic changes first seen on ultrasound as early as eight weeks [47], PMD cases are typically detected between 13 to 20 weeks of gestation once the placental cysts progressively enlarge, becoming more numerous and complex as gestation proceeds [5,21,48]. In some cases, a gradual reduction in the size of placental vesicular lesions has been encountered [7,48]. While the edematous villous stroma (hydropic villi) in molar pregnancy causes the appearance of cysts, cysts in PMD are dilated vessels with varying degrees of flow, corresponding to degrees of color, leading to a “stained-glass” appearance [50].

The PMD cysts have low or absent color Doppler signals during the first two trimesters. In contrast, in the third trimester, underneath and at the level of the chorionic plate, we can observe broad vascular zones with a turbulent blood flow with either arterial or venous blood [48]. Further, color Doppler helps to differentiate between PMD and other placental abnormalities with similar sonographic vesicular aspects: chorioangioma (large vessel/increased vascularity), spontaneous abortion with hydropic changes (no vessels), molar pregnancy (high velocity with low resistance flow), complete mole with coexisting normal fetus (the lesion affects the entire placental thickness, the cysts lack blood flow signal, the lesion is beyond the fetal sac), subchorionic hematoma (no vessels), and partial hydatidiform mole [48,51,52]. Three-dimensional (3D) ultrasound reconstruction usually demonstrates a multicystic placental mass with cysts that do not communicate with each other, have various diameters and are separate but adjacent to a normal-appearing placenta [49]. The ultrasound evaluation is crucial to early detection and proper management of PMD with IUGR [48], as well as other anomalies such as BWS, that occur in 21–30% of PMD cases, hepatic mesenchymal hamartoma, or other complications (omphaloceles, fetal anemia, and thrombocytopenia, facial or pulmonary hamartoma) [7]. The prenatal differential diagnosis between PMD and complete hydatiform mole with a twin live fetus (CHMTF) could be delineated with MRI acquisition [13]. The MRI evaluation and blood oxygen level-dependent MRI technique may provide an alternative imaging modality to diagnose the alteration of the entire placenta, or to measure the placental blood flow and volume during fetal growth, or changes in fetal and placental oxygenation [44,53]. The advantage of the MRI investigation includes high-quality images that are less dependent on maternal body habitus and may be obtained even in cases in which oligohydramnios is present [44]. The indication of MRI during pregnancy, even after the first trimester, should be carefully considered because safety has yet to be established [13].

The ultrasonographical, gross, and pathological similarities between PMD and partial hydatidiform mole often cause their misdiagnoses. The outcomes and treatment of the two are different, so the differential diagnosis must be adequately established [5,44].

For differential diagnosis, PMD is distinguished from partial and complete mole by ultrasonographic and histopathological examination. In partial molar pregnancy, the ultrasound findings show a thickened placenta, with multicystic, hypoechoic areas, and an abnormal fetus [7,15,28]. In contrast, PMD has in addition to the similar appearance of hypoechoic, multicystic areas, areas of the normal placenta, an apparently normal fetus [28]. Complete molar pregnancy with co-Twin shows a multicystic placenta with hypoechoic areas in one gestational sac and a normal placenta in the other sac [52]. The fetus associated with the normal placenta is apparently normal. There may or may not be a separating membrane between the two placentas [15]. Gross histopathological analysis reveals a large for gestational age placenta, with cystically dilated vesicles in all the three distinct pathologies [41]. As for microscopic analysis, PMD and Complete molar pregnancy with co-Twin show dilated stem vessels [42,48]. Partial molar pregnancy shows less prominent dilated stem vessels. Trophoblastic proliferation occurs only in the partial and complete molar pregnancies and not at all in PMD [15].

The PMD placenta is unusually large for the gestational age, with a relatively long tortuous marked twisted umbilical cord [47]. On gross examination, changes in the villi can be observed, with their morphology varying by gestational age, suggesting that the vascular malformations develop progressively: from chorionic plate non-dilated vessels and the normal and abnormal poorly delineated areas to aneurysmally dilated and tortuous vessels [47,51]. In some cases, discrete chorangiomas and extramedullary hematopoiesis can arise. These modifications are the impact of placental hypoxia [51].

The histological transformations in PMD include cistern formation with lax connective tissue, enlarged stem villi [4], and the most important clue for the differential diagnosis from partial hydatiform mole, the lack of trophoblast proliferation, and the absence of stromal trophoblastic inclusions [4,6]. As mentioned before, changes in the PMD placenta vary by gestational age: at the pregnancy inception, the stem villi display dilated cisterns encircled by loose myxomatous stroma, which contains sheer vessels under the trophoblastic layer, while in the third trimester, changes comprise enlarged thick-walled vessels in the chorionic plate, with fresh or mature thrombi that obstruct the arterial and venous lumina, and fibromuscular hyperplasia [51]. Irrespective of the pregnancy age, the terminal villi may also exhibit mesenchymal cell hypercellularity and stromal fibrosis, similar to the stem villi [51].

On immunohistochemical stains, some patterns may be encountered [44]. The p57kip2 protein is the only maternal gene [49]. Since villous cytotrophoblastic cells of complete molar pregnancy lack maternal genome, antibodies against p57kip2 protein may be a potential marker in distinguishing PMD from molar pregnancy, being negative for this test [49,51]. The immunohistochemical detection in PMD shows androgenetic/ biparental mosaicism in stromal cells; the mosaicism is uncommon in molar pregnancies [47,53,54]. Both the normal and dysplastic villi are positive for desmin and vimentin; however, smooth muscle actin is typically expressed in normal villi and is absent in dysplastic villi [10,44,51]. The dysplastic villi lack the increases in Ki-67, which translates to low proliferative rates [44]. The cells in PMD that line the cistern are negative for D2–40 and CD34, arguing against a lymphatic or vascular origin, respectively [44,53]. In our case, the negative CD-34 immunohistochemical stain in the cells that line the cistern, the normal p57 expression in the trophoblast, but absent stromal staining in placental mesenchymal dysplasia, the p53 protein expressed in the nuclei of some trophoblastic cells (p53 wild-type pattern), and the intense positive cytokeratin 7 staining of the villous trophoblastic cells, settled the diagnosis.

Chromosomal aberrations may appear in some fetuses, but most are phenotypically and karyotypically normal females [51]. Arizawa and Nakayama [11] incriminate two genes for the development of PMD, the overexpression of VEGF-D (related to angiogenesis) and paternally imprinted IGF2 (related lymphangiogenesis in vitro). An alteration on the X chromosome on the locus of the VEGF-D gene (Xp22.31) might explain the high occurrence of PMD in female fetuses [10,11]. The presence of cholangitis, fibroid hyperplasia, and lymphangiogenesis in PMD is compatible with the theory that altered gene expression may modify the regional hormonal sensitivity and conduct to discrepancies in the placental villus mesenchymal phenotype [25]. DNA ploidy and karyotyping can be performed utilizing flow cytometry, image analysis, cytogenetics, and fluorescent in situ hybridization on fresh or fixed tissue [51]. DNA ploidy may not help differentiate PMD from complete moles and spontaneous abortions with hydropic changes because most of them will be diploid [51]. It is recommended to exclude an abnormal karyotype, thus partial molar pregnancy [1]. In PMD, the fetal karyotype is usually normal (biparental, diploid, with 1 set from each parent and 46 chromosomes), whereas the PMD placenta is usually mosaic. The membranes, chorionic mesoderm, and vessels are diandric diploid (46 paternally chromosomes). The trophoblastic cells have normal biparental diploidy [16], but fetal aneuploidy is not excluded [30]. Biparental/androgenetic mosaicism is rarely diagnosed in humans. It is typically prenatally ascertained based on PMD, with fetal outcomes ranging from intrauterine demise to term-delivery IUGR [55]. Repnikiova et al. published the first case of a liveborn male neonate with biparental/androgenetic mosaicism noticed in the placenta, concomitant with other tissue (toe mass). The infant associated PMD, thrombocytopenia, anemia, soft tissue overgrowth on his fifth toe, hemangiomas over his foot, right buttock and chest, hepatic mesenchymal hamartoma, and congenital hypothyroidism, whose later development was in ranges [55].

In our case, the CVS detected two genetic profiles at the level of the placenta, one with maternal and paternal normal alleles and one with paternal alleles only.

The cause of PMD is yet unknown, current theories sustaining the idea of either an egg fertilized by two spermatozoa or a maternal nondisjunction error while the first division of a unique ovum and sperm [12]. The latter theory is the one that is more likely to be close to the truth since PMD lacks trophoblastic proliferation and stromal trophoblastic inclusions [4,6]. In maternal nondisjunction, the first meiotic division results in one normal cell with both maternal and paternal genes and one with paternal genes only; after additional divisions, these androgenic cells are confined to the placental vessels and membranes in a mosaic pattern interspersed with normal biparental cells [12,56]. The biparental cells from the first meiotic division continue to divide and mature normally, eventually forming the trophoblastic layer that develops into a significant portion of the placenta [12,56]. In contrast, the excessive proliferation of the trophoblastic cells from the molar placenta contains an abnormally elevated amount of paternal DNA [12]. In complete molar pregnancies, the trophoblastic cells lack maternal DNA, whereas partial moles include maternal and paternal DNA with two sets of paternal chromosomes. Instead of the single set found in normal cells and PMD [12].

PMD is associated with Beckwith–Wiedermann syndrome (BWS) in 25% of cases [57]. Abnormal expression of the imprinted genes on 11p15.5 may result in BWS. It includes epigenetic mutations at one of two imprinting centers (loss of methylation at the differentially methylated region 2 (DMR2) or gain of methylation at DMR1 on the maternal chromosome), a mutation in the maternal copy of CDKN1C (which encodes the protein p57kip2), as well as a paternally imprinted gene expressed from the maternal allele. Moreover, insulin-like growth factor 2 (IGF2), predominantly expressed from the paternal allele or paternal duplication or mosaic paternal uniparental disomy involving the entire imprinted region in 11p15.5, might play a role [10,30,57,58].

Most of the reported cases of PMD are singleton pregnancies, but there are documented cases of one twin PMD placenta either from monochorionic or dichorionic twin pregnancy [16,17,27]. The newborns were both male and female, had IUGR, were delivered prematurely in 2/3 cases [16,17,27], and experienced IUFD in one case [27].

Phenotypically normal fetuses associated with PMD might also encounter complications throughout the pregnancy, such as prematurity due to IUGR or IUFD [1,51]. In PMD cases with no proof of Beckwith-Wiedemann syndrome, 50% of the fetuses have IUGR and 43% undergo intrauterine or neonatal death, and 9% develop gestational hypertension, preeclampsia, eclampsia, or HELLP syndrome [12]. Severe IUGR and preeclampsia are the consequence of hypoperfusion and hypoxia. This happens due to the diversion of the fetal blood within the vascular abnormalities, thrombosis of the blood vessels within the stem villi, or related to chorioangiomas [10,20,23,51]. As noticed by Jauniaux et al. [23], placental size has little influence on the clinical outcome, while the complications in cases with chorioangioma are related more to the vascularity of the tumor rather than its size. Chen et al. [22] reported 2 PMD cases with chorioangioma, one neonate presenting thrombocytopenia and anemia, the other suffering from haemangiomatosis. Neonatal hematological conditions such as anemia and thrombocytopenia may be secondary to microangiopathic hemolytic anemia because of abnormal blood shunting and consumption in the dilated vessels [9,18,51,59]. PMD associated-fetal complications are not widely recognized by obstetricians and pediatricians. As a result, hematological complications among neonates are underestimated [59]. Since the mesenchymal cells in the villi, chorangiomas, haemangiomas, and chorionic vessels are all derived from the mesoderm, one can conclude that PMD, malformation of the placental vascularization, fetal haemangiomas, and chorangiomas, may represent a composite form of inborn malformation of the mesoderm [22].

Because about one-fourth of the PMD cases associate BWS, some of the encountered complications in fetuses born from PMD pregnancies are secondary to BWS without fully developed phenotypic traits [51]. For instance, hyperinsulinemic hypoglycemia witnessed in PMD neonates is secondary to pancreatic islet cell hyperplasia, a frequent finding in BWS [14,51].

The PMD newborns with normal phenotype should be followed-up for BWS features of the mesenchymal tumor (hepatic mesenchymal hamartoma, congenital adrenal hyperplasia, and vascular hamartoma) [51]. Some cases of PMD are diagnosed in conjunction with mesenchymal hamartoma of the liver (MHL), while in others, the diagnosis is made after an MHL diagnosis [24,59]. MHL is a benign hepatic tumor characterized by excessive, focal growth of an admixture of epithelial and vascular components, which becomes multicystic as it enlarges, providing a poorer prognosis than for fetuses without structural anomalies (IUFD or neonatal death) [54,59]. Therefore, obstetricians may need to perform detailed placental ultrasound studies to identify this PMD complication when a fetal liver tumor is found [59].

In contrast to a molar pregnancy, PMD is not associated with malignant trophoblastic tumors and carries no indications for pregnancy termination [12]. Intrauterine screening techniques for PMD and its complications include a blend of imaging methods (2D and 3D ultrasound, biophysical profile, Doppler assessment), clinical evaluation (uterine fundal height), genetic assessment (karyotyping), and serum markers for PMD (MSAFP) and gestational hypertension/preeclampsia/HELLP syndrome (maternal blood pressure, 24 urine screen for protein, complete blood cell count, liver function tests, creatinine, lactate dehydrogenase, coagulation studies) [12]. If carefully monitored, women with PMD pregnancies can deliver a healthy neonate [12]. Most normal-appearing infants did not show any developmental problems at the follow-up [51]. Mothers with PMD pregnancies showed no sign of repetition of PMD in subsequent pregnancies at the 5-year follow-up [19,51]; however, 15% of BWS cases are familial, so a PMD recurrence in such families is probable [51]. Sun et al. [5] reported a case of PMD and alive female neonate, whose follow-up results showed that, despite the lack of trophoblastic anomaly at pathologic examination, the patient developed choriocarcinoma in the smooth muscle of the uterine scar five months after cesarean section and a single metastatic nodule appeared in the left lung seven months after cesarean section. The patient underwent chemotherapy, the lung nodule gradually narrowed and disappeared after six courses of chemotherapy, with favorable evolution of the patient. The authors ask themselves, given the experience with their case of PMD, if follow-up protocol of PMD patients should include close monitoring for human chorionic gonadotrophin after delivery or hysterectomy after delivery [5]. This association between placental mesenchymal dysplasia and choriocarcinoma cannot establish a link. There are reported cases of choriocarcinoma in normal pregnancies, without fetal, placental, or maternal problems, that have developed choriocarcinoma [60,61,62].

## 6. Conclusions

This paper seeks to draw attention to the prenatal diagnosis of placental mesenchymal dysplasia. Due to its rarity, compared to a molar pregnancy, this pathologic condition with a poorly understood etiology often tends to be confounded with a molar pregnancy. The two diseases have a significantly different outcome for both mother and the fetus, in favor of PMD. Doctors tend to terminate the pregnancy in fear of the potentially unpleasant consequences of molar pregnancy. Since most cases with proper antenatal care have a satisfactory outcome, this is not recommended in PMD cases. When confronted with an enlarged multicystic placenta, the greatest challenge is the correct diagnosis of proper management. When faced with a patient with suspected PMD, doctors must explain the associated risks, including the in utero fetal death. Doctors should also suggest to pathologists the suspicion of PMD diagnosis in cases of enlarged placentas.

## Figures and Tables

**Table 1:** Literature review for case reports and case series of PMD; placental and biochemical findings.

Nr.	Author	Year	Number of Cases	Karyotype	Elevated Alpha-Fetoprotein	Elevated Human Chorionic Gonadotrophin	Placenta Weight	Immunohistochemical Staining
1.	Guenot et al. [1]	2019	5/22 *	Chromosomal evaluation of the 6 infants revealed no SA	5 (23%)	3 (14%)	NA	NA
2.	Moscoso et al. [2]	1991	2	46XX, 46XX No SA	>2.5 MoM	Normal	1200 g 3.03 MoM 3280 g 7.92 MoM	AFP and factor VIII were negative
3	Li et al. [3]	2014	1	46XX Chromosomal evaluation of the infant revealed no SA	Elevated (6.22 U/mL)	16 GW normal level 4335 mUI/mL 3 days postpartum 7.5 mUI/mL 3 weeks after delivery	760 g	negative for CD34 and D2-40 vimentin labeled p57kip2 negative low detectable Ki-67 expression
4.	Sun et al. [5]	2020	1	46XX No SA	NA	NA	520 g	positive p57 in all cytotrophoblast cells of PMD low detectable Ki-67 expression in PMD
5.	Toru et al. [6]	2014	1	NA No SA	NA	NA	487 g	negative for CD34 and D2-40
6.	Rosner–Tenerowicz et al. [8]	2020	1	46XX No SA	Elevated	Normal	500 g (90th percentile for GW)	NA
7.	Ishikawa et al. [9]	2016	1	46XX No SA	22 MoM (7261 ng/mL)- 23 GW 33 MoM (10,786 ng/mL)-30 GW 4990 ng/mL–3rd day postpartum	NA	575 g	NA
8.	Pham et al. [10]	2006	5/11 *	5 of 46XX No SA	NA	10.300 IU/L	686 g 2.38 MoM 670 g 1.76 MoM 450 g 1.14MoM 440 g 1.06 MoM 1000 g 2.42 MoM	No detectable Ki-67 or Flk-1 protein expression in either tissue
9	Arizawa and Nakayama [11]	2002	6/15 *	6 of 46XX No SA	NA	NA	685 g 940 g 440 g 950 g 860 g 930 g	NA
10.	Adams et al. [12]	2018	1	46XY No SA	3,8 MoM	48.000 IU/L	NA	NA
11.	Himoto et al. [13]	2014	3	3 of 46XX No SA	NA	90.436 mIU/mL, 98.171 mUI/mL, 151.370 mUI/mL	1100 g 930 g 838 g	NA
12.	Chan et al. [14]	2003	1	46XY No SA	NA	NA	513 g	NA
13	Woo et al. [15]	2011	1	46XX No SA	NA	Elevated-167402 mUI/mL, 6.95 MoM	1380 g	P57 stain decreased, but not absent diploidy
14	Gheysen et al. [16]	2018	2/1 **	Monochorionic diamniotic twin pregnancy 46XY/46XY No SA	NA	NA	NA	Stem villi: the stromal fibroblasts were p57 negative whereas the trophoblastic cells were p57 positive
15	Jitsumori et al. [17]	2018	2/1 **	Dichorionic diamniotic twin pregnancy 46XX/ 46XX No SA	NA	24 GW – 44,084 mIU/mL	Both placentas: 1066 g (above the 90th percentile)	p57kip2 was lost in the PMD lesions
16	Sander et al. [18]	1993	3	46XY, 46XX,46XX No SA	NA	NA	600 g (1.45 MoM) 815 g (2.52 MoM) 829 g (2.09 MoM)	NA
17.	Matsui et al. [19]	2003	1	46XX No SA	NA	65,960 mUI/mL 27 GW	465 g (>90% of the normal range for GW)	NA
18.	Hojberg et al. [20]	1994	1	46XX No SA	3.03 MoM 15 GW	47,000 mUI/mL 15 GW	1500 g	NA
19.	Ohira et al. [21]	2013	1	46XX No SA	NA	241,270 mIU/mL at 12 GW, and then gradually decreased	1200 g	NA
20.	Chen et al. [22]	1997	2	46XX, 46XX No SA	NA	NA	1150 g 1100 g	NA
21.	Jauniaux et al. [23]	1997	3	46XX, 46XX, 46XX No SA	NA	NA	1535 g 1430 g 1250 g	NA
22.	Huang et al. [24]	2021	1	46XX No SA	4.57 MoM 15 GW	12 MoM 15 GW 60,000–160,000 mUI/mL	1350 g	NA
23.	Heazell et al. [25]	2009	1	46XX No SA	N	90,376 mUI/L 13 GW	NA	Mib-1 (Clone Ki-67) anti-CD34 anti-cytokeratin 7 and anti-E-cadherin
24	Lee et al. [26]	1990	1	46XX No SA	NA	NA	1490 g	NA
25	Kaiser-Rogers et al. [27]	2006	3/2 **	Case 1: Dichorionic twin placenta Case2: Dichorionic twin placenta 46XY, 46XX No SA	Case 1: NA Case 2: 1.64 MoM in 15 GW	Case 1: NA Case 2: Elevated 5.09 MoM in 15 GW	Case 1: 1900 g Case 2: 690 g	NA
26	Psarris et al. [28]	2020	1	46XX No SA	NA	free β hCG was 33.77 IU/L (0.83 MoM) serum PAPP-A was 3.790IU/L (1.587 ΜοΜ)	720 g	NA
27	Pal et al. [29]	2017	1	46XX No SA	485 ng/mL	25,780 mIU/L	950 g (20 × 20 × 3 cm)	NA
28	Toscano M.P. and Schultz R. [30]	2014	1	46XX No SA	NA	NA	1415 g (28.0 × 25.0 × 7.0 cm)	NA
29	Gizzo et al. [31]	2012	1	46XX 11GW chorionic villus sampling normal female karyotype	NA	Normal values	1100 g Increased thickness (6 cm)	NA
30	Balachandran et al. [32]	2015	1	NA No SA	NA	NA	600 g	NA
31	Taga et al. [33]	2013	1	46XX No SA	NA	20124.97 U/L at 20 GW (normal)	720 g 20 × 16 × 2 cm	NA
32	Qichang et al. [34]	2013	1	46XX No SA	NA	4611 mUI/mL at 2 days postpartum undetectable at 3 weeks postpartum	1370 g 30 × 25 × 4.5 cm the largest tumor measured 11 × 8 × 4.5 cm	Expression of p57^KIP2^ in the villous cytotrophoblast
33	Koga et al. [35]	2014	1	46XX No SA	NA	NA	1690 g 25 cm in diameter	positive for vimentin and desmin, loss of p57
34	Gibson et al. [36]	2004	1	46XY No SA	NA	NA	1258.0 g 23.0 × 18.0 × 3.5 cm	NA
35	Kinoshita et al. [37]	2007	1	46XX No SA	NA	67,500 mIU/mL at 19 GW(normal)	930 g 21 × 19 × 5 cm	NA
36	Gurram et al. [38]	2016	1	46XX No SA	NA	NA	1970 g (>95th percentile)	NA
37	Mulch et al. [39]	2006	1	46XX No SA	Elevated MSAFP (2.9 MoM at 18 GW)	NA	480-g placenta, 3.5 × 1.8 × 1.9 cm	NA
38	Surti et al. [40]	2005	2/1 **	Twin gestation Twin A 46XX Twin B 46XY No SA	NA	NA	Diamniotic dichorionic 1325 g twin placenta Placenta A:370 g Placenta B: 955 g	NA
39	Rohilla et al. [41]	2012	1	46XX No SA	NA	Postpartum after 3-weeks-0.01 IU/dl	1700 g	NA
40	McNally et al. [42]	2021	3	46XX 46XY 46XX No SA	amniocentesis: abnormal secondary to elevated AFP	The third case-the highest value of 72,786 IU/L	NA	NA
41	Reed et al. [43]	2008	1	46XX No SA	NA	NA	893 g; (expected weight 316 g)	p57KIP2 immunoreactivity

Legend 1. SA—specific abnormalities, NA–not available, MoM—multiple of the median, GW—gestational weeks, PAPP-A—pregnancy-associated plasma protein A; * the total number of healthy newborns among all reported cases of placental mesenchymal dysplasia, by the respective author; ** total number of healthy newborns from twin pregnancies with placental mesenchymal dysplasia.

**Table 2:** Literature review for case reports and case series of PMD; Fetal outcomes and complications during pregnancy.

Nr.	Author	Year	Number of Cases	Preterm Delivery	Complications of the Mother: Preeclampsia/Gestational Hypertension, Gestational Diabetes, etc.	Fetal Outcome	Uncomplicated Pregnancy
1.	Guenot et al. [1]	2019	5/22 *	9/14 (64%)	6 (27%)	11 (50%) IUGR	3 (14%)
2.	Moscoso et al. [2]	1991	2	36 GW CS 37 GW CS	NO	2200 g 2940 g	YES
3	Li et al. [3]	2014	1	35 GW VD pPROM	NO	1800 g APGAR 10/10 Pathological jaundice 30 GW–IUGR (<10th percentile) Follow-up of neonate and mother-uneventful at 10 months	YES
4.	Sun et al. [5]	2020	1	37 GW CS for IUGR	IUGR	2290 g APGAR 10/10, jaundice Follow-up results-trophoblastic dysplasia in the uterine scar 5 months after cesarean section; the morphology was consistent with choriocarcinoma.	NO
5.	Toru et al. [6]	2014	1	32GW CS for intractable maternal tachycardia	atrial-mitral valve replacement operation used warfarin from the first trimester, no history of fever (with or without rash)	2550 g; healthy baby First-trimester screening for aneuploidy had revealed a risk of 1:780 for Down syndrome The baby and mother were discharged in good condition 2 weeks later	NO
6.	Rosner-Tenerowicz et al. [8]	2020	1	29 GW pPROM ECS	NO	1320 g APGAR 5/6 (74th percentile) Pathological CTG 80 mm multifocal liver cyst- surgery in the 4th day -simple cyst of the liver Good outcome	YES
7.	Ishikawa et al. [9]	2016	1	30 GW	NA	Transient tachypnea Neonate anemia 8.3 g/dL Normal findings on brain MRI at 93 days	YES
8.	Pham et al. [10]	2006	5/11 *	30–37 GW	NA	2-IUGR 1 Severe pallor and hypotonia; neonatal anemia and thrombocytopenia 2 normal newborns	2
9	Arizawa and Nakayama [11]	2002	6/15 *	24–38 GW	NA	6 normal newborns	NA
10.	Adams et al. [12]	2018	1	33 GW CS	NA	AP 5/8, 1600 g (7th percentile), IUGR Decreased FHR reactivity and late decelerations	NA
11.	Himoto et al. [13]	2014	3	39 GW VD 40 GW VD 33 GW ECS	1 gestational diabetes	2- IUGR 1- OLIGOAMNION 1- FETAL DISTRESS	1
12.	Chan et al. [14]	2003	1	36 GW iVD	Mild preeclampsia	2195 g	NO
13	Woo et al. [15]	2011	1	33 GW PPROM VD	Preeclampsia	1802 g, APGAR 4/7	NO
14	Gheysen et al. [44]	2018	2/1 **	34 GW CS	Hyperthyroidism (Treatment: propothiouracil)	Twin 1 2130 g APGAR 8/9 Twin 2970 g APGAR 7/9, severe IUGR, severe oligoamnios	NA
15	Jitsumori et al. [17]	2018	2/1 **	32 GW +5 days CS	NO	Twin 1–1799 g APGAR 7/8 Twin 2–1215 g, APGAR 8/9, IUGR)	YES
16	Sander et al. [18]	1993	3	NA	NA	2183 g (5th percentile)—IUGR 1985 g (60th percentile)—thrombocytopenia 2356 g (25th percentile) Normal	NA
17.	Matsui et al. [19]	2003	1	27 GW CS	Placenta praevia with massive bleeding	820 g (within 50% of the normal range for gestational age) APGAR 1/2	NO
18.	Hojberg et al. [20]	1994	1	At term VD	NO	2860g	YES
19.	Ohira et al. [21]	2013	1	39 GW ECS	NO	1998 g (<3rd percentile) APGAR 8/9 Prolonged decelerations in FHR monitoring	YES
20.	Chen et al. [22]	1997	2	37 GW VD 27 GW VD pPROM	Polyhydramnios (both cases)	1500 g, APGAR 5/9; hemangiomatosis (face, left year auricle, left arm, both palms, hepatic hemangioma-surgical removed) and hepatic cyst 976 g, APGAR 6/6, anemia (7,9g/dL) Both cases: follow-up 1 year–good outcome	NO
21.	Jauniaux et al. [23]	1997	3	39 GW VD 40 GW VD 37 GW VD	NO	2400 g 3650 g 3320 g Normal findings at 1-year follow-up	YES
22.	Huang et al. [24]	2021	1	36 GW V	NO	2626 g APGAR 9/10	YES
23.	Heazell et al. [25]	2009	1	38 GW CS	Oligohydramnios	2700 g (8th percentile)	YES
24	Lee et al. [26]	1990	1	36 GW ECS	Partial placenta praevia	2001 g, respiratory distress, anemia 5.6 g/dL Good outcome for mother and infant	NO
25	Kaiser-Rogers et al. [27]	2006	3/2 **	Case 1: 34 GW, Case 2: 37 GW iVD for IUGR	Case 1: Twin 1 IUFD Case 2: IUGR	Case 1: Twin 1 severe IUGR, IUFD, liver cyst Twin 2: normal growth and development (58thpercentile) Case 2: Twin 1 normal boy 2942 g APGAR 10/10 Twin 2 normal girl breech extraction 2210 g APGAR 10/10, IUGR (<5th percentile)	NO
26	Psarris et al. [28]	2020	1	36 GW CS severe IUGR	Severe IUGR	2210 g APGAR 9/10	NO
27	Pal et al. [29]	2017	1	37 GW VD	NO	2450 g APGAR 8/9	NO
28	Toscano M.P. and Schultz R. [30]	2014	1	36 GW VD	NO	2230 g (16thpercentile, −0,98 z score, adequate for gestational age), APGAR 9/9, Jaundice Good outcome for mother and infant	YES
29	Gizzo et al. [31]	2012	1	36 GW iVD for severe IUGR coexistent with itching and cholestasis of pregnancy	increased factor IX and factor XI (thrombosis prophylaxis) hypothyroidism pulmonary embolism during contraceptive therapy itching and cholestasis of pregnancy	2100 g APGAR 7/8/9 The baby was in good health, with no external dysmorphic features. She had transient physiological neonatal jaundice without other complications.	NO
30	Balachandran et al. [32]	2015	1	40 GW VD	At 8GW—vaginal bleeding	2250 g Post-natal follow-up was normal up to 12 weeks	YES
31	Taga et al. [33]	2013	1	37 GW CS for previous CS	NA	2520 g APGAR 8/9 Postoperative course was uneventful	YES
32	Qichang et al. [34]	2013	1	27^+3^ GW (preterm labor) VG—unsuccessful tocolysis	Polyhydramnios AFI- 37.5 cm	740 g APGAR 5/8	NO
33	Koga et al. [35]	2014	1	37 GW ECS non-reassuring fetal status and fetal growth restriction	NA	1812 g APGAR 1/6, IUGR Anemic, with a bleeding tendency (Hemoglobin, 6.4 g/dL); Blood film showed a leukoerythroblastic picture with circulating myelocytes, nucleated red cells, schizocytes at 1 week of age, no bleeding tendency no other underlying disease and hematologic complications did not recur	NO
34	Gibson et al. [36]	2004	1	39 GW VD	NA	3250 g vascular hamartoma of the face and abnormal eye movements (left eye ptosis) which was repaired	YES
35	Kinoshita et al. [37]	2007	1	39 GW CS due to non-reassuring fetal state	NA	1452 g female (<10th centile) severe IUGR	NO
36	Gurram et al. [38]	2016	1	38 GW VD	NA	2700 g infant, born in good condition, discharged home day 2 postpartum liver harmartoma - planned to be operated in 1 year	YES
37	Mulch et al. [39]	2006	1	36 GW VD induced labor after amniocentesis for fetal lung maturity	NA	2800 g APGAR 8/9 No abnormalities have been found in the infant during a follow-up period of 1 year	YES
38	Surti et al. [40]	2005	2/1 **	35 GW CS discordant twin growth reversal diastolic flow within the umbilical artery of Twin B	NA	Twin A—a female, weighed 2312 g Twin B—a male, weighed 1603 g At 18 months of age, both the twins showed normal development, although Twin B continues to be slightly smaller than his twin sister A	NO
39	Rohilla et al. [41]	2012	1	36 GW VD pPROM tachycardia of the fetus 180 beats/min mild oligohydramnios	febrile (38.5°C) MPR 94/min	2450 g APGAR 8/9, IUGR At 3-years old, the child had normal development	NO
40	McNally et al. [42]	2021	3	39 GW 36 GW 29 GW ECS HELLP	HELLP in the third case	No fetal anomalies	YES YES NO
41	Reed et al. [43]	2008	1	30 GW CS fetal distress	NA	1110 g (expected: 1280 ± 350 g) At 11 months of age, cystic liver mass-resection.	NO

Legend 2. GW–gestational weeks, CS—cesarean section, ECS—emergency cesarean section, VD—vaginal delivery, iVD—inducted vaginal delivery, pPROM—Preterm premature rupture of the membranes, APGAR—Appearance, Pulse, Grimace, Activity, and Respiration, HELLP—Hemolysis, Elevated Liver enzymes and Low Platelets syndrome, FHR—fetal heart rate, MPR—maternal pulse rate, IUGR—intrauterine growth restriction, IUFD—intrauterine fetal death, CTG—cardiotocography, MRI—magnetic resonance imaging, NA—not available; * the total number of healthy newborns among all reported cases of placental mesenchymal dysplasia, by the respective author; ** total number of healthy newborns from twin pregnancies with placental mesenchymal dysplasia.
